# Helpfulness of Question Prompt Sheet for Patient-Physician Communication Among Patients With Advanced Cancer: A Randomized Clinical Trial

**DOI:** 10.1001/jamanetworkopen.2023.11189

**Published:** 2023-05-02

**Authors:** Joseph Arthur, Varsha Pawate, Zhanni Lu, Sriram Yennurajalingam, Ahsan Azhar, Akhila Reddy, Daniel Epner, David Hui, Kimberson Tanco, Marvin Omar Delgado Guay, Marieberta Vidal, Minxing Chen, Eduardo Bruera

**Affiliations:** 1 Department of Palliative, Rehabilitation and Integrative Medicine, The University of Texas MD Anderson Cancer, Houston; 2 Department of Biostatistics, The University of Texas MD Anderson Cancer Center, Houston

## Abstract

**Importance:**

There are few robust evaluations of disease-specific question prompt sheets (QPS) in patient-physician communication among patients with advanced cancer.

**Objective:**

To compare the patient perception of helpfulness, global evaluation, and preference for the QPS vs a general information sheet (GIS), and to examine the effect of the QPS on participants’ anxiety, participants’ speaking time, number of questions asked, and length of the clinical encounter.

**Design, Setting, and Participants:**

This controlled, double-blind randomized clinical trial was conducted at an outpatient palliative and supportive care clinic in a cancer center in the US. Eligible patients were 18 years or older, had a cancer diagnosis, and were undergoing their first outpatient consultation visit with a palliative care physician from September 1, 2017, to May 31, 2019. Data analysis used a modified intention-to-treat design. Data were analyzed from May 18 to June 27, 2022.

**Intervention:**

QPS, a 25-item list of questions developed by expert palliative care clinicians using a Delphi process and tested among ambulatory advanced cancer patients. The control was GIS, generic information material given routinely to patients seen at the supportive care clinic.

**Main Outcomes and Measures:**

The main outcome was patient perception of helpfulness. Secondary outcomes included global evaluation and preference of QPS compared with GIS immediately after the encounter.

**Results:**

A total of 130 patients (mean [SD] age, 58.6 [13.3] years; 79 [60.8%] female) were randomized to receive either QPS (67 patients [51.5%]) or GIS (63 patients [48.5%]). Patients considered QPS and GIS equally helpful, with no statistically significant difference (mean [SD] helpfulness score, 7.2 [2.3] points vs 7.1 [2.7] points; *P* = .79). The QPS group, compared with the GIS group, had a higher global positive view of the material (mean [SD] global perception score, 7.1 [1.3] vs 6.5 [1.7]; *P* = .03) and felt it prompted them more to generate new questions (mean [SD] rating, 7.0 [2.9] vs 5.3 [3.5]; *P* = .005). Of 47 patients asked their preference between the items, more participants preferred the QPS to the GIS in communicating with their physicians (24 patients [51.1%] vs 7 patients [14.9%]; *P* = .01) at the 4-week follow-up. No significant differences between the QPS and GIS groups were observed regarding participant anxiety, speaking time, number of questions asked, or consultation length (eg, mean [SD] anxiety rating, 2.3 [3.7] vs 1.6 [2.7]; *P* = .19).

**Conclusions and Relevance:**

In this randomized clinical trial, participants perceived both QPS and GIS as equally helpful, but they had a more positive global view of and preferred the QPS. QPS facilitated generation of new questions without increasing patient anxiety nor prolonging the consultation. The findings provide support for increased adoption and integration of QPS into routine oncologic care.

**Trial Registration:**

ClinicalTrials.gov Identifier: NCT03287492

## Key Points

**Question:**

What is patients’ perception of the helpfulness of a question prompt sheet (QPS) compared with a general information sheet (GIS) for patient-physician communication?

**Findings:**

In this randomized clinical trial of 130 patients with advanced cancer, participants perceived both QPS and GIS as helpful, but they had a more positive global view of and preferred QPS to GIS in communicating with their physician. QPS reportedly facilitated the generation of new questions without increasing patient anxiety or prolonging the consultation visit.

**Meaning:**

These findings provide support for increased adoption and integration of QPS into routine oncologic care.

## Teaser

This randomized clinical trial compares patient perceptions of a question prompt sheet vs a general information sheet for facilitating patient-physician communication among patients with advanced cancer.

## Introduction

A question prompt sheet (QPS) is a structured list of potential questions that are available for patients to ask physicians during a clinical encounter. It may allow practitioners to meet patients’ desired information needs, assist with decision-making, and improve the overall communication process.^1^ This is vital because patients sometimes are unsure about the questions to ask their physicians, forget to ask the relevant questions, or feel uncomfortable to ask certain questions.^2,3^ A QPS may also prevent physicians from conveying unsolicited and potentially distressing information to patients.^4^ Studies have demonstrated the value of a QPS in patient-physician interactions in diverse fields of medicine.^5,6,7,8,9,10,11,12^

However, there is insufficient data regarding the utility of a QPS among patients with advanced cancer.^13,14^ Moreover, very few methodologically robust evaluations of a QPS in a head-to-head comparison with an attention control group have been conducted. The main objective of this study was to compare patients’ perceptions about the helpfulness, overall global evaluation, and preference for a systematically developed QPS vs a standard general information sheet (GIS) during patient-physician encounters. We also examined the effect of the QPS on participants’ anxiety, participants’ speaking time, the number of questions asked, and the length of the clinical encounter.

## Methods

### Study Design, Participants, Procedures

This randomized clinical trial was approved by the institutional review board of the University of Texas MD Anderson Cancer Center, Houston. All participants provided written informed consent. The trial protocol and statistical analysis plan are available in Supplement 1. The study followed the Consolidated Standards of Reporting Trials (CONSORT) reporting guideline. This trial was conducted among patients seen at the outpatient Palliative and Supportive Care Clinic at the University of Texas MD Anderson Cancer Center from September 1, 2017, to May 31, 2019. This clinic sees patients with advanced cancer who are referred by their primary oncologists for the management of complex physical, psychosocial, and spiritual needs, as well as assistance with medical decision-making and overall goals of care. Eligible patients were aged at least 18 years, had a cancer diagnosis, were undergoing their initial outpatient consultation visit with 1 of 10 palliative care physicians, and could read and communicate in English. After providing written informed consent, patients completed baseline questionnaires and were then randomly assigned in a 1:1 fashion to receive either the QPS or the GIS 30 minutes prior to their physician consultation. Randomization was conducted by the biostatistician via the institution’s clinical trial conduct website using the Pocock-Simon method. Patients were stratified by physician to carefully control for physicians’ impact on the primary end point. Both interventions were concealed in identical opaque envelopes. Patients, research staff who enrolled the patients, and physicians were blinded to the study assignments. Patients were encouraged to read the information material before the visit. Physicians were asked to endorse the use of the information material during the encounter by asking the patient if they had any questions, and either explaining why it was important to ask questions or inviting the patient more than once to ask questions.^5,15^

Conversations were audiotaped and later transcribed. At the end of the consultation, patients completed questionnaires assessing their views about the information material they received, their overall satisfaction with the consultation, and their anxiety level. The participating physicians also completed a physician assessment questionnaire. In an exploratory open-label format, patients who returned for follow-up at 4 weeks (±7 days) openly received both the QPS and the GIS 30 minutes prior to seeing their physician and were encouraged to use the materials in preparation for their visit. After the visit, they indicated which of the materials they preferred.

### Data Collection

Patients’ demographic and clinical characteristics were obtained from their medical records. Race and ethnicity were categorized as Asian, Black, Hispanic or Latino, White, and other (including American Indian or Alaskan Native, refused to answer, and unknown). Race and ethnicity were included in analyses because we wanted to explore any potential association between the use of the communication aids and those variables. The deidentified audio recordings were transcribed by a professional medical transcription company. The number and types of questions that patients asked were carefully and independently extracted from the transcribed data by one experienced investigator (V.P.) and then verified by a second investigator (J.A.); any discrepancies were discussed in detail until a mutual agreement was reached.

### Study Interventions

The QPS (eAppendix 1 in Supplement 2) is a single-page list of 25 questions that was developed by an expert panel of clinicians using a Delphi process^16^ and later tested for its content validity among a group of patients and caregivers attending an ambulatory palliative medicine clinic.^17^ The GIS (eAppendix 2 in Supplement 2) is a single page of generic informational material that was created by our group and is routinely provided to patients who are seen at the clinic. It contains general patient information about palliative care and other related information felt to be relevant to new patients.

### Questionnaires and Outcome Measures

The primary outcome, patients’ perception of helpfulness, and other views about the information materials were assessed immediately after the consultation using the Patient Assessment Questionnaire. This is a 7-item, 0- to 10-point scale that assessed the extent to which patients felt the material helped them to communicate with their physician, was clear or easily understandable, had the right amount of information, would be recommended to other patients, did not make them anxious, helped them to think of questions or concerns they had not previously thought of, and would be used in the future. The mean score across all the 7 individual patient ratings was calculated to obtain the global perception score, with higher score indicating more positive perception. The questionnaire has been used in several previous studies.^6,17,18^ Patients’ satisfaction with the consultation was assessed using the Patient Satisfaction Questionnaire,^15,19,20^ a 5-item visual analogue scale ranging form 0 to 100, with an internal reliability (Cronbach α) of 0.90 and higher score indicating more satisfaction.^20^ Patient anxiety was measured by the Spielberger State Anxiety Inventory, a 20-item self-report scale^21^ with high reliability (*r* = 0.93), internal consistency, and validity.^21^ Scores range from 20 to 80, with higher score indicating greater anxiety. Baseline patient preferences for information were measured using 2 items from the Cassileth Information Styles Questionnaire, with 1 item consisting of a 5-point Likert scale that assessed the amount of detail a patient preferred (1 indicates very little; 5, as much as possible) and the other item a multiple choice question asking what kind of information a patient preferred, with options “I want only the information needed to care for myself properly,” “I want additional information only if it is good news,” and “I want as much information as possible, good and bad.”^22^ Baseline patient preferences for level of involvement in decision-making were assessed with the validated Control Preferences Scale.^23,24,25^ Overall preference for the QPS or GIS was assessed using a single multiple-choice question: “Now that you have had the opportunity to use the two different information materials, overall, which of them would you prefer to use in communicating with your doctor?” Patients could select whether they preferred either material a little or a lot more, or whether they had no preference. The Physician Assessment Form asked physicians to indicate on a scale of 0 to 10 points their perception about the helpfulness of the information material to the patient, its effect on the visit duration, and their overall satisfaction with the consultation, with higher score indicating more positive perception. Other outcome measures included the total number and types of participant questions, speaking times, and overall consultation duration.

### Statistical Analysis

The primary outcome was patients’ perception of helpfulness (0-10 scale) of the informational material. A 2-sample *t* test was applied to examine the outcome difference between the QPS and the GIS group. With 136 enrolled patients and a 5% attrition rate, we estimated 80% power to detect a difference in means of 2 on a 0- to 10-point scale of the primary outcome, assuming an SD of 4 using the 2-sample *t* test with a 2-sided significance level of *P* = .05. Summary statistics, such as means and SDs, were used to describe continuous variables, while frequencies and percentages were used to describe categorical variables. Similar 2-sample *t* and χ^2^ tests or Fisher exact test were used to examine the group difference for selected secondary outcomes. Goodness-of-fit test was used for χ^2^ to assess patients’ overall preference after using both information materials concurrently. Associations of the demographic or clinical factors with the primary outcome were assessed using ordinary least-squared regression. Analysis was modified intention-to-treat because 5 randomized patients (3.7%) who did not receive the allocated intervention and had missing data were excluded. *P* = .05 was used to determine the statistical significance for all secondary outcomes analyses, given this portion of the analyses was exploratory and was for hypothesis generating purpose. Data were analyzed using Stata/SE version 16.1 (StataCorp). Data were analyzed from May 18 to June 27, 2022.

## Results

A total of 135 eligible patients were randomly assigned to receive either the QPS or GIS. After excluding the 5 randomized patients (3.7%) who did not receive the allocated intervention, data were available for 130 patients (mean [SD] age, 58.6 [13.3] years; 79 [60.8%] female), including 67 patients (51.5%) randomized to GPS and 63 patients (48.5%) randomized to GIS (Figure). There were no significant differences in the baseline demographic and clinical characteristics between the 2 groups. (Table 1)

**Figure.**
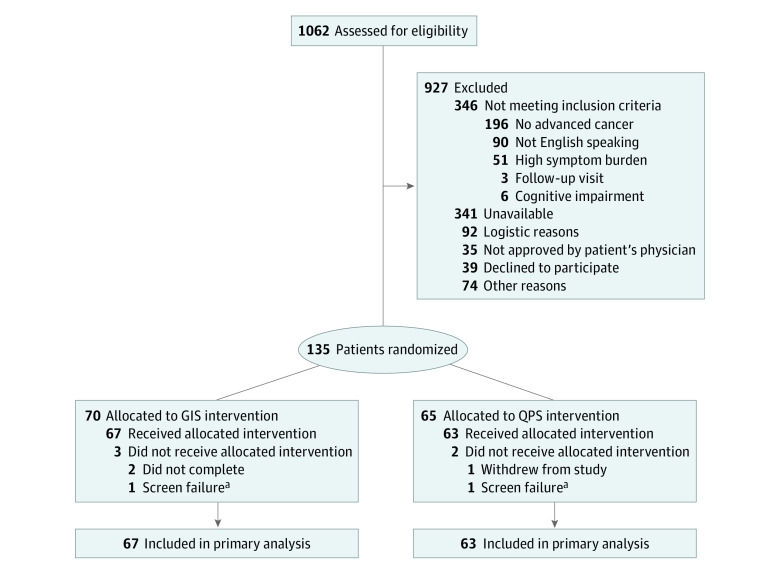
Flowchart of Patients Enrolled in the Study GIS indicates general information sheet; QPS, question prompt sheet. ^a^Patient was found to be ineligible after randomization.

**Table 1.** Baseline Demographic and Clinical Characteristics of Included Patients

Characteristic	No. (%) (N = 130)	
	QPS group (n = 63)	GIS group (n = 67)
Age, mean (SD), y	57.5 (13.9)	59.61 (12.8)
Sex		
Male	20 (31.7)	31 (46.3)
Female	43 (68.3)	36 (53.7)
Race and ethnicity		
Asian	6 (9.5)	1 (1.5)
Black	3 (4.8)	7 (10.4)
Hispanic or Latino	2 (3.2)	7 (10.4)
White	51 (81.0)	52 (77.6)
Other^a^	1 (1.6)	0
Cancer type		
Breast	13 (20.6)	9 (13.4)
Gastrointestinal	13 (20.6)	20 (29.9)
Genitourinary	6 (9.5)	9 (13.4)
Gynecologic	8 (12.7)	2 (3.0)
Head and neck	11 (17.5)	11 (16.4)
Thoracic	6 (9.5)	6 (9.0)
Other	6 (9.5)	10 (14.9)
ECOG performance status		
0-2	44 (71.0)	50 (74.6)
3-4	18 (29.0)	17 (25.4)
Missing	1	0
Presence of a caregiver		
Yes	44 (69.8)	49 (73.1)
No	19 (30.2)	18 (26.9)
Patient preferences decision-making		
Active	13 (20.6)	5 (7.5)
Collaborative	43 (68.3)	50 (74.6)
Passive	6 (9.5)	8 (11.9)
Do not know or prefer not to answer/no information	1 (1.6)	4 (6.0)
Total baseline anxiety score, mean (SD)^b^	46.7 (7.4)	46.72 (8.2)
Patient preferences for information, mean (SD)^c^	4.7 (0.8)	4.32 (1.2)
Symptoms, mean (SD)^d^		
Pain	4.8 (3.0)	4.7 (3.0)
Fatigue	5.6 (2.8)	4.8 (3.0)
Nausea	2.5 (2.9)	1.8 (2.7)
Depression	2.0 (2.8)	2.0 (2.5)
Anxiety	2.5 (2.8)	2.6 (3.0)
Drowsiness	2.7 (2.6)	2.9 (2.9)
Shortness of breath	2.1 (2.8)	2.1 (2.8)
Appetite	4.5 (2.9)	3.7 (2.8)
Well-being	4.4 (2.6)	4.0 (2.7)
Sleep	4.8 (2.7)	4.5 (2.7)
Total Symptom Distress Score	35.8 (16.7)	33.2 (17.8)

Abbreviations: ECOG; Eastern Cooperative Oncology Group; GIS, general information sheet; QPS, question prompt sheet.

^a^ Other includes American Indian or Alaskan Native, patient refused, and unknown.

^b^ Measured using the Spielberger State Anxiety Inventory. Scores range from 20 to 80, with higher score indicating higher anxiety.

^c^ Measured using the Cassileth Information Styles Questionnaire, with a range of 1 to 5, with higher score indicating more desire for information.

^d^ Measured using the Edmonton Symptom Assessment System, with a range of 0 to 10, with higher score indicating more severe symptom intensity. Total score is the sum of all 10 items, with a range of 0 to 100.

Perception of helpfulness was equally high, with no statistically significant difference between the QPS and the GIS groups (mean [SD] helpfulness score, 7.2 [2.3] points vs 7.1 [2.7] points; *P* = .79). The QPS prompted participants to think of new questions more than the GIS did (mean [SD] score, 7.0 [2.9] vs 5.3 [3.5]; *P* = .005). Participants had a higher global perception score for the QPS than the GIS (mean [SD] score, 7.1 [1.3] vs 6.5 [1.7]; *P* = .03) (Table 2). All 47 participants who returned for their 4-week follow-up appointment participated in the open-label phase. The demographic and clinical characteristics of patients who returned and those who did not were not significantly different, including age, race, cancer type, type of intervention received at the initial visit, and Edmonton Symptom Assessment System (ESAS) total Symptom Distress Score. Therefore, the informative missingness of the data was largely ignorable. After using both informational materials concurrently, more participants preferred the QPS to the GIS in communicating with their physicians (24 patients [51.1%] vs 7 patients [14.9%]; no preference: 16 patients [34.0%]; *P* = .01) (Table 3). In a separate analysis, there were no differences in the effects of the QPS and GIS on physicians’ perceptions of the helpfulness (mean [SD] score, 6.79 [2.74] vs 6.27 [2.96]; *P* = .32), the consultation length (mean [SD] score, 8.33 [2.53] vs 8.52 [2.14]; *P* = .67), or overall satisfaction (mean [SD] score, 8.74 [1.38] vs 8.72 [2.06]; *P* = .95).

**Table 2.** Patient Views of the Information Material

Patient view of information material	Mean (SD)^a^		*P* value
	QPS group (n = 63)	GIS group (n = 67)	
Helpful in communication	7.2 (2.3)	7.1 (2.7)	.79
Clear to understand	8.8 (1.4)	8.5 (1.8)	.21
Right amount of information	8.5 (1.6)	8.1 (1.9)	.18
Will recommend to others	8.0 (1.9)	7.8 (2.3)	.61
Will use similar material in future	7.6 (2.6)	7.6 (2.6)	.91
Made me less anxious	2.3 (3.7)	1.6 (2.7)	.19
Prompted me to think of new questions	7.0 (2.9)	5.3 (3.5)	.005
Global perception score^b^	7.1 (1.3)	6.5 (1.7)	.03

Abbreviations: GIS, general information sheet; QPS, question prompt sheet.

^a^ Measures are on a scale from 0, indicating least, to 10, most.

^b^ Indicates the mean score across all of the 7 patient views on the information material.

**Table 3.** Patients’ Overall Preference After Using Both Information Materials Concurrently During the 4-Week Follow-up Visit

Preferred information material	No. (%) (n = 47)		*P* value^a^
	Expected	Observed	
Question prompt sheet	15.6 (33.3)	24.0 (51.1)	.01
General information sheet	15.6 (33.3)	7.0 (14.9)	
No preference	15.6 (33.3)	16.0 (34.0)	

^a^ χ^2^ Goodness-of-fit test.

The mean physician speaking time was not significantly different between the 2 groups (eTable in Supplement 2). Participants in the QPS group spoke less than those in the GIS group (mean [SD] time, 8.0 [5.3] minutes vs 10.0 [5.3] minutes; *P* = .06). Both groups asked more treatment-related questions and fewer prognosis- and end-of-life–related questions. No significant association was observed between the QPS and the GIS groups regarding the number and types of questions asked. Overall, both groups were equally satisfied with the consultation. (mean [SD] score, QPS: 95.01 [10.51] vs GIS: 93.90 [14.18]; *P* = .63). Patients’ change in anxiety scores from baseline were also similar in both groups (mean [SD] anxiety rating, 2.3 [3.7] vs 1.6 [2.7]; *P* = .19).

Table 4 shows the factors associated with participants’ perception of helpfulness the information material they received. Compared with White patients, Black and Hispanic patients were significantly more likely to perceive either of the informational materials they received as helpful (coefficient, 1.95; 95% CI, 0.72 to 3.18; *P* = .002). In addition, older age (coefficient, 0.04; 95% CI, 0.01 to 0.07; *P* = .02) and lower ESAS depression (coefficient, −0.20; 95% CI, −0.38 to −0.01; *P* = .04) were associated greater perceived helpfulness of the informational material.

**Table 4.** Factors Associated With the Perception of Helpfulness of the Information Material Patients Received^a^

Factor	Coefficient (95% CI)	*P* value
Age, y	0.03 (−0.01 to 0.06)	.10
Male sex	−0.77 (−1.64 to 0.10)	.08
Race and ethnicity		
Black	2.03 (0.38 to 3.68)	.02
Hispanic or Latino	2.04 (0.33 to 3.76)	.02
White	0 [Reference]	NA
Other^b^	−3.54 (−7.94 to 0.86)	.11
ECOG performance status (Reference: ≤2)	0.98 (0.03 to 1.93)	.04
ESAS score		
Anxiety	−0.15 (−0.33 to 0.03)	.10
Drowsiness	0.14 (−0.03 to 0.31)	.12
Well-being	−0.19 (−0.37 to −0.01)	.04

Abbreviations: ECOG, Eastern Cooperative Oncology Group; ESAS, Edmonton Symptom Assessment System; NA, not applicable.

^a^ Ordinary least squares regression stepwise estimation; variables with *P* value <.20 were considered in building the model. Information material indicates either question prompt sheet or general information sheet.

^b^ Other includes American Indian or Alaskan Native, patient refused, and unknown.

## Discussion

In this randomized clinical trial, patients perceived both the QPS and GIS as helpful when communicating with their physician, with no significant difference between groups. However, patients felt the QPS facilitated generation of new questions. They also had a better overall global view of the QPS, and after using both materials concurrently during a follow-up visit, patients preferred the QPS to the GIS for communicating with their physicians. Previous studies by our group have reported the perceived helpfulness of the QPS during patient-physician communication.^17,18^ In a randomized clinical trial comparing a disease-specific QPS with a GIS among 60 women with breast cancer consulting with their medical oncologists, we found that patients perceived the QPS as more helpful than the GIS.^18^ Although participants in this study perceived both materials as helpful, their better global view of and relative preference for the QPS validate its value in routine clinical care and further underscore the need for its integration in clinical guidelines and health policies. The use of a GIS as an attention control group in this study allowed for a more rigorous and robust evaluation of the QPS. Only a few studies have compared the QPS with another communication aid. Moreover, data on the focal evaluation of patients’ perceptions about QPS’ utility are limited.

The QPS did not increase patient anxiety during the clinical encounter. This should reassure health care practitioners who may be concerned that the QPS questions will be emotionally upsetting and negatively impact patients’ psychological outcomes. Several studies have examined the association between the use of a QPS and patient anxiety.^5,6,7,17,26,27,28,29,30^ Many did not find any significant association with anxiety,^5,6,7,26^ while a few studies showed a decrease in patient anxiety levels^17,27^ immediately after,^28^ and 6 weeks,^29^ and 4 months,^30^ after initial the consultation. A study by Brown et al^26^ randomized 318 patients with cancer consulting with their oncologists to either receive or not receive a QPS and found that QPS patients whose physicians passively responded to questions from the QPS had higher anxiety than did those whose physicians proactively addressed questions from the QPS and controls.

We found that the QPS neither prolonged the duration of the visit nor increased the physician or patient speaking time. In fact, participants in the QPS group spoke less than did those in the GIS group, suggesting that the QPS may improve the efficiency of communication without prolonging clinical encounters. Previous studies by our group and others also observed no association of the QPS with consultation length.^17,18,31,32,33,34^ In a randomized clinical trial of 174 patients with advanced cancer who were assigned to receive either the QPS or standard consultation without QPS, Clayton et al^5^ found that QPS consultations were longer than controls, probably because a longer 20-page QPS brochure consisting of 112 items was used in that study.^5^ It is conceivable that such observation was not found in this study because we used a disease-specific, single-page 25-item QPS. Future studies are needed to investigate the effect of QPS length on consultation duration.

Although patients felt the QPS facilitated generation of new questions, it did not result in an increase in the number of questions asked. The goal with the use of a QPS is to empower patients to generate and ask essential questions that meet their information needs. The QPS may effectively improve communication quality without necessarily increasing the number of questions that patients ask. Patients may be able to ask their most meaningful questions rather than simply asking more questions. In that regard, patient self-report of the helpfulness of the material might be a highly reliable indicator of benefit from the information material. Further studies are needed to ascertain the best means of measuring the true utility of the QPS.

Compared with previous findings,^5,31,35^ patients in this study asked more treatment- and symptom-related questions and fewer prognosis and end-of-life–related questions. This may be because a considerable number of them were still receiving disease-directed therapy and therefore had a particular interest in treatment- and symptom-related questions and concerns. In clinical settings, such as the inpatient palliative care units where patients have more advanced disease, prognosis and end-of-life questions might be more relevant. Moreover, patients might have preferred to first focus on their acute issues and would eventually discuss the more sensitive prognosis and end-of-life issues once their acute physical symptoms were addressed and they had the opportunity to build a closer therapeutic relationship with their physicians.

The reason why Black and Hispanic patients were more likely to perceive the information material as helpful is unclear, but it suggests that a written material that aids in patient communication might be particularly valued by members of racial and ethnic minority groups, including Black and Hispanic patients. In a different study, the QPS was found very acceptable by Black patients with cancer and effectively increased their active participation in racially discordant interactions.^36^ Similarly, our findings also suggest that an informational material may be particularly useful to older patients in guiding them to navigate important conversations with their physicians.

Major medical organizations, such as the National Cancer Institute, the National Academy of Medicine, and the American Society for Clinical Oncology, have alluded to the benefits of good communication in quality of care and emphasized the need for improved patient-physician communication among patients with advanced illnesses.^37,38,39,40^ The QPS is a simple, inexpensive tool that might help in achieving this goal. Despite increasing evidence regarding the utility of QPS in physician-patient consultations, it has not been fully adopted and implemented in oncologic settings. Some barriers to its full implementation include a feeling among patients of being overwhelmed by the sheer amount of written information.^41,42^ It is challenging to develop a universal QPS that suits all patients’ needs in view of the vast diversity within the population of patients with cancer and the dynamic nature of patient-physician communications. Wide variations in patient learning styles, communication goals, degrees of knowledge, and emotional capabilities may present real challenges in using a standardized QPS for all.^43^ One potential solution is to ensure that the development of a QPS is distinctively tailored to specific patient populations to enhance its efficacy. An electronic health system that integrates an interactive QPS that allows patients to generate their own list of questions based on their individual preferences and information needs would be ideal.^44^

### Limitations

This study has some limitations. One limitation is that it was conducted at a single tertiary academic center. Therefore, the results might not be generalizable to other clinical settings. In addition, we were unable to record the specific QPS questions that participants eventually asked during the visit. A better understanding of how participants used the material in real time and which questions were the most useful should be a focus in future research. Another limitation is that hypothesis testing for the secondary end points is considered exploratory when the primary end point does not show statistical significance, which was the case for this study. Additionally, the study was conducted among ambulatory patients with relatively good functional status. Future studies should include patients in acute inpatient settings, since they might have different symptom severity and therefore different outcomes.

## Conclusions

This randomized clinical trial found that patients perceived both QPS and GIS as equally helpful in communicating with their physician during consultation. However, they had a more positive global evaluation of, and preferred the QPS to the GIS. The QPS reportedly facilitated generation of new questions without increasing patient anxiety nor prolonging the consultation visit. The findings support the adoption, integration, and implementation of QPS in routine oncologic care.

## Notes

Supplement 1.
Trial Protocol and Statistical Analysis Plan

Supplement 2. **eAppendix 1.** Question Prompt Sheet **eAppendix 2.** General Information Sheet **eTable.** Effect of Information Material on Participant Speaking Time, Consultation Duration, Questions Asked, Anxiety and Overall Satisfaction

Supplement 3.
Data Sharing Statement
