# Supplementary material for: Helpfulness of Question Prompt Sheet for Patient-Physician Communication Among Patients With Advanced Cancer: A Randomized Clinical Trial
Source: JAMA Netw Open. 2023 May 2;6(5):e2311189. doi: 10.1001/jamanetworkopen.2023.11189 (PMC10155065; doi:10.1001/jamanetworkopen.2023.11189)
Supplement: Supplement 1. — Trial Protocol and Statistical Analysis Plan [file jamanetwopen-e2311189-s001.pdf]

Randomized Controlled Trial of a Question Prompt Sheet versus a General Information Sheet in Patients with Advanced Cancer

Principal Investigator: Dr. Joseph Arthur
Co-PIs: Dr. Sriram Yennu; Dr. Eduardo Bruera
Collaborators: Minxing Chen

Table of Contents

A. Objectives……………………………………………………………………………….…1
B. Hypothesis……………………………………………………………………………….…2
C. Background…………………………………………………………………………..….2
D. Rationale…………………………………………………………………………………3
E. Research Design……………………………………………………….………………4
F. Statistical Considerations……………………………………………..………………11
G. Data Confidentiality Procedures…………………………………………..…………13
H. References……………………………………………………………………..… ………14

A. OBJECTIVES

Primary

1. To compare patients' perception of helpfulness in communicating with their physicians between a question prompt sheet (QPS) and a general information sheet (GIS).

Secondary

2. To compare caregivers' perception of helpfulness in communicating with their physicians between the QPS and the GIS.

3. To examine the level of patients' and caregivers' overall satisfaction with the consultation with the use of the QPS versus the GIS.

4. To examine physicians' views about the information material and overall satisfaction with the use of the QPS versus the GIS.

5. To explore how the use of QPS affects the average speaking time of patient or physician during the consultation visit.

6. To establish demographic and clinical predictors of patients' perception of helpfulness of a QPS

7. To examine the overall patients' preference between the QPS and GIS in an open label phase.

8. To compare the change in patient anxiety state with the use of the QPS versus the GIS.

9. To explore the factors underlying patients' preferences for the QPS or GIS.

B. HYPOTHESIS

Patients will perceive the use of a question prompt sheet as more helpful compared with a general information sheet in communicating with their physicians.

C. BACKGROUND

Patients with advanced disease develop peculiar physical, psychological, social and spiritual needs that require a multidimensional approach to address them[1] and an effective communication strategy becomes the fundamental backbone of this holistic intervention. Approaches that encourage patients to actively participate and ask appropriate questions during their visit are important to enhance their understanding of their illness and empower them to make important decisions regarding their medical care. Studies have shown that greater patient participation during physician-patient encounters has been linked with positive patient psychological outcomes.[2-4] However, patients and family members may be uncertain about what type of questions to ask their physicians, may forget to ask certain pertinent questions, or may feel too embarrassed to ask them.[5,6] Similarly, physicians may be unsure about the type and extent of information to provide their patients, when to convey them, or whom to have those conversations with.[7,8] Situational factors, such as change in disease status may alter a patient's preferences for information and involvement.[9] The use of precise communication interventions might therefore help to address some of these barriers.

Examples of strategies used to improve physician-patient communication include the provision of

an audio recording[10,11], a post encounter summary letter to the patient after the clinic visit[11-14],

providing patients with decision aids[15], coaching via interactive media or face-to-face[5], and the use of a prompt sheet.[10,16] A prompt sheet is a structured list of potential questions available for the patient to ask the physician during a clinical encounter. It may help patients to obtain their desired level of information regarding their illness, assist with decision making, and facilitate the overall communication process.[17] It may also prevent physicians from conveying unwanted information to patients which can potentially increase their level of distress.[18]

Various versions of a prompt sheet have been developed in different fields of medicine including general medicine,[19] geriatric medicine,[20] gynecological and dermatological conditions,[21] surgical patients,[22] diabetes, [23] and cancer. [10] Results from their use have been variable but predominantly positive. Some studies on the use of a prompt sheet in cancer settings showed that they were well accepted by patients and providers[24] and increased the number and specific types of questions asked during clinical encounters[25, 26]. On the other hand, Butow et al. in a randomized control trial of 142 cancer patients receiving either a prompt sheet or general information sheet found that the prompt sheet did not increase the number of questions asked and the patient speaking time.[27]

Prompt sheets are not regularly used in advanced cancer or outpatient palliative care. As integration of palliative care into routine oncologic care increases, ways to ensure that patients are able to ask questions that are important to them have become more relevant. Clayton et al. in a thoroughly conducted study developed a comprehensive booklet that contained questions addressing various topics in palliative care.[26] However, one limitation was that it was about 20 pages long containing 112 questions, and therefore impractical for routine use at a busy outpatient clinic with time constraints and where the need for brevity is paramount in the delivery of care. Hebert et al. developed and tested a question prompt among family caregivers of cancer patients and physicians regarding discussion on end-of-life issues and had positive outcomes[28]. However, the study did not include the patients themselves. Yeh et al. administered a question prompt sheet to 30 advanced cancer patients prior to their initial outpatient clinic consultation and found that 90% of the patients perceived the sheet as helpful and relevant. The study was however limited by the lack of an attention control group[29].

D. RATIONALE

The use of a question prompt list has been shown to improve physician-patient interaction during clinical consultations.[25,26,29] However, there is paucity of data on its use in the palliative care setting.[28] In an initial study, our team developed a consensus list of prompt sheet questions in a Delphi process by a panel of 21 palliative care experts in the Department of Palliative Care and Rehabilitation Medicine at the University of Texas M. D. Anderson Cancer Center[30]. In a follow up preliminary study, we tested the sheet for its content validity in a group of patients and families and had positive patient reported outcomes. The next step is to conduct a randomized control trial comparing the QPS with a general information sheet among advanced cancer outpatients.

E. RESEARCH DESIGN

E1. Study design and setting

The study is a randomized-controlled trial to be conducted at the outpatient Supportive Care Center at the University of Texas MD Anderson Cancer Center. We plan to enroll 136 patients and up to 136 caregivers. Up to 20 physicians will be enrolled. Each physician may see up to 15 patients in this study.

E2. Participant selection

Patient participation

Inclusion criteria

1. Age 18 years or older
2. First outpatient consultation visit with a palliative care specialist.
3. Normal cognitive status, defined as a normal state of arousal and an absence of obvious clinical findings of confusion, memory deficits or concentration deficits, as determined by the patient's physician.
4. Ability to read and communicate in English
5. Diagnosis of advanced cancer
6. Signed written informed consent form.

Exclusion criteria

1. Refusal to participate in the study

Caregiver participation

A caregiver will be eligible for the study if he/she: i) accompanied the patient to the clinic visit, ii) is identified by the patient as someone who is actively involved in their overall care, iii) is able to read and communicate in English, iv) is willing to participate in the study and able to complete the questionnaires.

Physician participation

A physician will be eligible if he/she is i) a palliative medicine specialist, ii) seeing the patient in consultation on the day of the study, iii) willing to participate in the study.

E3. Randomization

Participating patients will be randomized in a double-blind fashion into one of two treatment groups: the active group will receive the QPS intervention and the control group will receive the GIS intervention. Randomization will be conducted via the Clinical Trial Conduct website developed by the Department of Biostatistics. We will randomize patients using Pocock-Simon's method and the randomization will be stratified by "physician" in order to carefully control for physicians' impact on patients' perception of helpfulness (primary endpoint).

E4. Blinding

Both the QPS and GIS will be concealed in opaque envelopes that will be identical to each other. The research staff and the patient/ caregiver will be blinded to the contents of the envelopes. The physician will also be blinded to the type of written material given to the patient. We successfully conducted this blinding protocol in a similar previous study[16].

E5. Research team

An orientation training will be organized for all research team members involved in the study in order to equip them with the necessary knowledge and expertise for the conduct of the study and also to standardize the provision of each intervention.

Figure 1. Summary of Study Design

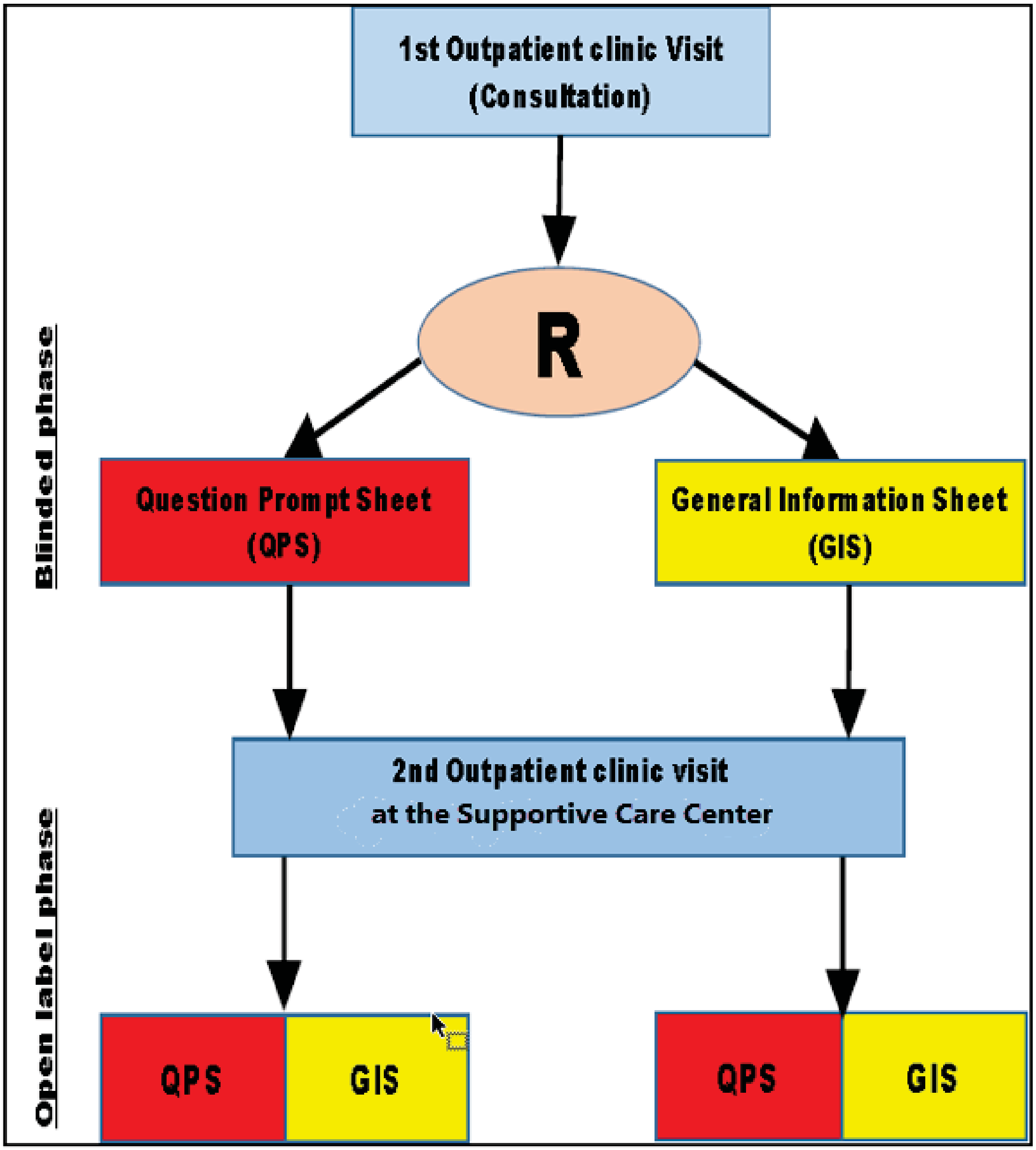

E6. Study Procedures

The research staff will first identify a potentially eligible candidate by screening all scheduled patient consultations on that day, and then inform and obtain a verbal consent from the attending physician for the patient's participation in the study. After the routine nurse assessment, the

research coordinator will obtain patient's written consent and then ask him/ her to complete the Spielberger State Trait Anxiety Inventory ***(Appendix A)***, 2 questions from Cassileth Information Styles Questionnaire ***(Appendix B)***, and the Control Preferences Scale ***(Appendix C)***. The patient will then be randomized to receive either the QPS ***(Appendix D)*** or the GIS ***(Appendix E)***. It will take approximately 5 minutes to read through the QPS or GIS. The questionnaires should take about 5 minutes to complete. Caregivers accompanying patients to the clinic who are willing to participate in the study will also be asked to provide consent and will be given the same information material (either QPS or GIS). In a situation where more than one eligible caregiver is present, the one most involved in the patient's care, as determined by the patient, will be the first person to be approached. If he/she declines to participate, the next most involved caregiver identified by the patient will then be approached until there are no more eligible caregivers present.

During the patient-physician encounter, the physician will be asked to endorse question asking. Physician endorsement of question asking will be defined according to the following standardized protocol: 1) The physician asks the patient/ caregiver if they have any questions; and either 2) explains why it is important to ask questions, or 3) invites the patient/caregiver to ask questions more than once.[31,32] The conversation will be recorded with a voice recorder and later transcribed. The audio-recordings will be sent via Box.com to Landmark Associates Inc. to be transcribed professionally. At the end of the consultation, the research staff who will be absent during the conversation, will ask the patient to complete the Spielberger State Trait Anxiety Inventory ***(Appendix A)*** again, the questionnaires assessing patients'/caregivers' views about the information material ***(Appendix F)*** and overall satisfaction with consultation ***(Appendix G).*** The questionnaires should take about 13 minutes to complete. The participating caregiver will also separately complete ***Appendix F***. It should take about 5 minutes to complete. All the information materials will be collected from the participants after the consultation but those who indicate the desire to keep them will be given a copy of their forms for future use. At the end of the study, all study patients will receive a patient information booklet which also contains the GIS content. This is part of usual practice in the clinic and it will ensure that all patients get to receive identical standardized clinical care. The physician will also be asked to complete a physician assessment questionnaire ***(Appendix H)*** after the clinic encounter. This should take about 2 minutes to complete.

In a subsequent open label phase to be conducted during their next follow up clinic visit at the Supportive Care Center (SCC), patients who participated in the initial blinded study will now openly receive both information materials prior seeing the physician. At the end of the visit with the physician, patients will complete a questionnaire assessing their overall preference between the two information materials. ***(Appendix I)*** Patients who are unable to complete the questionnaire in the clinic they will be contacted over the phone within 48 hours to complete the questionnaire. The phone call will be audio-recorded. Patients will not be provided copies of this recording. This should take approximately 2 minutes to complete.

Using a qualitative methodology, we will also conduct a descriptive exploratory repeat assessment to gain a broad understanding of patients' preferences for the information materials they receive prior to their doctor visit for the purpose of improving physician-patient communication. A sub-setof 20 patients in this study will be invited to participate in individual interviews after their follow up visits. We will purposively sample to get a range of ages, genders, ethnicities, and disease types for the interviews and match these characteristics in both treatment groups. The individual interviews will be conducted in person in a private clinic room after the physician visit by an interviewer trained in qualitative techniques (see Appendix J for interview guides). Patients who are unable to complete the interview in person will be contacted over the phone within 48 hours and requested to complete. It is anticipated that each interview will last approximately 20 minutes. At the end of each interview, the interviewer will record a field note of the circumstances and events during the interview. All interviews will be digitally recorded and professional transcribed by Landmark Associates Inc.for analysis.

E7. Data Collection and coding

The research nurse/research coordinator will review the patient's chart and collect information regarding diagnosis, demographics, and other patient characteristics (such as patient age, gender, race, cancer diagnosis, Edmonton Symptom Assessment System [ESAS] scores). The demographic and clinical characteristics will be obtained from either the patients' medical records or from the participant responses to the study assessment questionnaires that will be administered during the study. The transcribed consultation will be coded and pertinent information will be extracted by 2 trained coders who will be blinded to group allocation. The extracted information will include the type and category of each question asked such as the palliative care team/services provided, symptoms and treatment, prognosis, end of life issues, or other. Each coder will code all the transcripts and re-code a random 10% of their own initial work to determine intra-rater reliability,

and a random 10% of the other's work to determine inter-rater reliability.

E8. Study Interventions

Question Prompt Sheet (QPS) (Appendix D)

This is a single page, 25-item list of questions that was developed by an expert panel of clinicians in the Department of Palliative Care and Rehabilitation Medicine at the University of Texas M.D. Anderson Cancer Center using a 3 round Delphi process[30]. Some of the question were adapted from previous studies like the one by Clayton et al[26]. The QPS consists of some of the most relevant questions in diverse aspects of palliative care and is intended for use by patients and caregivers attending an outpatient palliative care clinic.

General Information Sheet (GIS) (Appendix E)

This is a locally developed sheet created by the faculty in the Department of Palliative Care and Rehabilitation Medicine at the University of Texas M. D. Anderson Cancer Center for routine use at the supportive care center. It contains general patient information about palliative care and other related information felt to be of high relevance to patients. The length and content of the GI sheet are easy for the patients and families to read during the waiting period before being seen by the physician and/or nurse.

E9. Outcome measures

1. **Patients' perception of helpfulness in communicating with their physicians between a question prompt sheet (QPS) and a general information sheet (GIS).**

This will be assessed as the proportion of patients who agree or strongly agree that the information was helpful in the communication with their physician. It will be measured using the third item of the **Patients/ Caregivers Assessment Questionnaire** consisting of rating scales regarding the information material **(Appendix F)**. Participants will rate the **perception of helpfulness** of the material using in a 5-point Likert scale from "strongly disagree" to "strongly agree" on item #3: "The material helped me to communicate with my doctor". It will also consist of 0 to 10 point scales assessing the extent to which the information material was helpful, was clear or easy to understand, had the right amount of information, was recommendable to other patients, did not make them anxious, enabled them to think of questions or concerns they hadn't thought of before, and a similar question sheet will be used by them before they see their doctor in the future. The questionnaire was developed from previous studies[16,29] and through discussions between the authors. (J.A; E.B).

2. **Caregivers' perception of helpfulness in communicating with their physicians**

between a question prompt sheet (QPS) and a general information sheet (GIS).

This will be assessed as the proportion of caregivers who agree or strongly agree that the information was helpful in the communication with their physician. It will be measured using item #3 of the **Patients/ Caregivers Assessment Questionnaire (Appendix F)**

We will also assess other patients' and caregivers' views about the information material by using the **Patients/ Caregivers Assessment Questionnaire (Appendix F)** using the mean scores of the following patient and caregiver views:

a. was helpful (0-10 scale)
b. was clear or easy to understand (0-10 scale)
c. had the right amount of information (0-10 scale)
d. was recommendable to other patients (0-10 scale)
e. did not make them anxious (0-10 scale)
f. enabled them to think of questions or concerns they hadn't thought of before (0- 10 scale)
g. a similar question sheet will be used by them before they see their doctor in the future (0-10 scale)

3. **Patients' and caregivers' overall satisfaction scores with the consultation with the use of between the QPS and the GIS.**

Patient satisfaction with the consultation will be assessed with the mean global satisfaction scores of the **Patient Satisfaction Questionnaire (PSQ)**[33-35] which consists of 5-items with 0 to 100 Visual Analogue Scale (VAS), 0 being "not at all" and 100 being "extremely" **(Appendix G)**. It measures patients' satisfaction with 1) how well their needs were addressed, 2) how actively involved they were in the interaction, 3) the adequacy of the information provided, 4) the adequacy of the emotional support provided, and 5) the overall consultation interaction. An overall satisfaction score is obtained by the average response on 5 items. The PSQ has been used to assess cancer patients' satisfaction with oncologist encounters in the inpatient and outpatient clinic settings[33,35]. Internal reliability (Cronbach's α) of the PSQ in one study was 0.90[35].

4. **Physicians' views about the information material and the consultation:**

Physicians' views about the information material and overall satisfaction will be assessed by the mean scores of 3 questions (0-10 scale) soliciting the opinion of the physician regarding 1) the helpfulness of the information material to the patient, 2) its effect on the visit duration, and 3) the physician's overall satisfaction with the consultation, using a 0 to 10 Likert Scale ***(Appendix H).***

5. **Effect of the QPS versus the GIS on the average speaking time of the patient or physician.**

This information will be extracted from the transcribed documents of the recording during the patient-physician clinical encounter. We will also measure the following outcomes from the same transcribed document:

a) Total duration of the visit in minutes;
b) Total number of questions asked by the patient and/or caregiver; and
c) Categories of each question asked, namely: the palliative care team/services provided, symptoms and treatment, prognosis, end of life issues, or other.

6. **Association between the patient perception of helpfulness and demographic (age, gender, ethnicity, educational level, presence of a caregiver) and clinical characteristics (cancer stage, pre-consultation anxiety level, patient preferences for information, and patient preferences for involvement in decision-making).**

a) Anxiety level will be measured by the ***Spielberger State Trait Anxiety Inventory (Appendix A)*** as described in Outcome #8 below.

b) Patient preferences for information will be assessed using 2 items from the **Cassileth Information Styles Questionnaire**[36] which assesses the amount of details and the type of information preferred by patients on a scale of 1 to 5. ***(Appendix B).***

c) Patient preferences for level of involvement in decision-making will be assessed using ***The Control Preferences Scale,*** a validated and reliable scale [37-40] that measures patients' preferred level of involvement in decision-making during consultations with their physicians ***(Appendix C)***. The tool consists of statements describing different decision-making roles and patients are classified as preferring 'active', 'passive', or 'collaborative' roles in making decisions regarding their treatment based on their choices to those statements.

7. **Overall patients' preference between the QPS and the GIS.**

This will be measured by the proportion of patients who would prefer to use the QPS over the GIS in communicating with their physicians after having the opportunity to use both materials. We will use a multiple choice question: *"Now that you have had the opportunity to use the two different information materials, overall, which of them would you prefer to use in communicating with your doctor?".* Patients will select their responses from 5 options ***(Appendix I).***

8. **The change in patient's anxiety state from pre-consultation to post-consultation.**

Patients' anxiety state will be measured using the ***Spielberger State Trait Anxiety Inventory (Appendix A).*** This inventory consists of two 20 item self-report versions which measure situational (state) and general (trait) anxiety. Each item consists of a 4-point Likert response scale. The state anxiety version will used in this study to measure participants' situational anxiety before and after the consultation visit[41]. Total score ranges from 20 to 80, where a score indicates greater anxiety. The inventory's simplicity makes it ideal for evaluating individuals with lower educational backgrounds. It's been found to have high reliability(r= 0.93), internal consistency, andvalidity[41].

E 10. Distress Plan

If any patient or caregiver experiences significantly high levels of distress while participating in the study, as per the clinical judgement of the palliative care physician, he/she will be taken off study and will be addressed by the physician, the palliative care counselor and/or other members of the interdisciplinary team as appropriate.

E11. Patient Safety and Monitoring

Patients will receive routine medical and nursing assessments from their assigned outpatient palliative care physician and clinical nurse during their visit in the clinic. In addition, our trained

research nurses will conduct study assessments and monitoring on the consultation day and the follow up day during the period when the study is being conducted. They will report any questions or concerns to the study physician who will be available by phone, email, or pager for further attention.

The principal investigator and the Institutional Review Board (IRB) will provide regulatory monitoring. Patient confidentiality will be ensured by use of unique study numbers, secure storage of clinical data, and anonymous reporting.

F.STATISTICAL CONSIDERATIONS

The primary outcome is patients' perception of helpfulness (0-10 scale) in communicating with their physicians after the first consultation visit (Appendix F, Question 4). At patient enrollment, randomization will be obtained via Clinical Trial Conduct website. A total of 136 patients will be randomized into 2 groups (QPS and GIS) in the ratio of 1:1 to reach 128 evaluable patients with 5% attrition rate due to withdrawal or lost to follow-up. With 128 evaluable patients, we will have 80% power to detect a difference in means of 2 assuming the common standard deviation of 4 using two group t-test with a two-sided significance level of 0.05. We will also reach out to patients' caregivers to collect information if available.

Standard statistics including mean, standard deviation, median, range, frequency and percentage will be summarized for variables of interest, such as patients' demographics, clinical characteristics, anxiety state, patients'/caregivers' views about the information material, patients' satisfaction, physicians' views about the information material and consultation, patients' preferences for information, patient preferences for level of involvement in decision-making and patients' overall preference between the QPS and the GIS at the follow-up visit. Two sample t- test or Wilcoxon rank sum test, whichever appropriate, will be used to compare continuous variables of interest between the QPS and the GIS. Chi-squared test or Fisher's exact test, whichever appropriate, will be used to test for associations between categorical variables and helpfulness of information material (helpful vs the rest) for each of the QPS and the GIS as well as the preference in types of information material (QPS vs GIS).

For the primary objective and secondary objectives 2, 3, 4, 8, two sample t-test or Wilcoxon rank sum test, whichever appropriate, will be used to compare a) patients'/caregivers' perception of helpfulness in communicating with their physicians, b) patients'/caregivers' overall satisfaction with the consultation, c) physician's view/overall satisfaction about the information material and d) the change in patient anxiety state scores before and after consultation between the QPS and the GIS. For the secondary objective 5, two sample t-test or Wilcoxon rank sum test, whichever appropriate, will be used to compare the average speaking time of patient or physician during the consultation visit. Also, general linear model will be applied to assess the effect of QPS on the average speaking time adjusting for clinically and/or statistically important factors. For the secondary objective 6, univariate/multicovariate logistic regression will be used to evaluate patients' demographic and clinical factors on patients' perception of helpfulness of QPS when dichotomizing the answer to the question 3 in Appendix F ("Agree/Strongly Agree"=Helpful vs the rest). For the secondary objective 7, we will estimate the preference of information material with 95% confidence intervals. Also, univariate/multicovariate logistic regression will be used to evaluate patients' demographic and clinical factors on overall patients' preference between QPS and GIS.

Descriptive exploratory analysis of both the physician-patient visit transcripts and patient post-visit interviews will be led by an experienced qualitative researcher (LW) using MAXQDA11 (VERBI GmbH, Berlin, Germany) qualitative analysis software. As the first 10 physician-patient visits are completed for each treatment group (total 20 visits), initial coding of question categories and themes will be done (LW). The analysis team will then meet (LW, JA) and question types and themes will be reviewed for agreement and understanding. Each team member (LW, JA) will then code 5 visit transcripts from each treatment group (total 20 visit transcripts). The team will then meet again to review coding to be certain there is agreement. If there is disagreement in the coding an additional 5 interviews per treatment group per team member (LW, JA) will be coded and the team will then meet to confirm agreement. This process will continue until agreement is consistent. The remainder of the interviews will be analyzed equally by treatment group and team member.

Initial coding of themes will be conducted (LW) for the post-visit interviews.

The analysis team (LW, JA) will then meet and review the coding. Changes will be made to the coding until team consensus is reached. Modifications and additions to the interview guide may be made based on the results of each time point analysis. When all analyses of post-visit interviews have been completed, the team will meet to integrate themes from each time point and across the project. A final description of themes of patients’ preferences for information to improve physician-patient communication will be developed.

Results of the analysis of the physician-patient visit transcripts and the patient post-visitinterviews will be compared to determine common themes and develop a final description of patient perception of helpfulness of the material in enhancing physician-patient communication.

Other statistical methods, when appropriate, may be applied. Since this study is of minimal risk to the patient, we will not conduct an intermediate analysis.

G.DATA CONFIDENTIALITYPROCEDURES

Health information will be protected and we will maintain the confidentiality of the data obtained from the database to the best of our ability.

Collection of identifiers: We will collect and securely store patients' identifiers (name and medical record number). Each patient will be assigned a study number that will be the only identifier to figure in the analytical file and personal data will not be disclosed in any form. The key linking these numbers will be retained in a securely locked file by the investigator.

Data Storage: Strict safeguards will be in place to protect participant privacy and data confidentiality. All electronic records will be stored on password-protected institution computers behind the institution firewall. Paper records will be stored in a locked cabinet in a locked office located in the Department of Palliative, Rehabilitation, and Integrative Medicine. Audiotapes will be digitally stored indefinitely and will include voice identifiers and study ID.

Training of personnel: Only MDACC personnel trained in maintaining confidentiality will have access to study records.

Data sharing: Landmark Associates Inc. will receive de-identified audio-recordings via Box.com to assist with professional transcription. Box.com is a password-protected content management and file sharing system behind the institution firewall.

Final disposition of study records: The data may be maintained indefinitely, aggregated in the future, and used for future research studies.

Audio recordings will be sent via secure and encrypted MD Anderson Outlook e-mail to participants upon request. We will utilize the encrypted e-mail method (“[send secure]”) to send the recordings to participants, as suggested by our Institutional Compliance Office.

H. REFERENCES

1. Arthur J, Bruera E: Supportive and palliative care: A poorly understood science for the perioperative clinician. Best Practice & Research Clinical Anaesthesiology 27:563-573, 2013
2. Kaplan SH, Greenfield S, Ware JE, Jr.: Assessing the effects of physician-patient interactions on the outcomes of chronic disease. Med Care 27:S110-27, 1989
3. Greenfield S, Kaplan S, Ware JE, Jr.: Expanding patient involvement in care. Effects on patient outcomes. Ann Intern Med 102:520-8, 1985
4. Gattellari M, Butow PN, Tattersall MH: Sharing decisions in cancer care. Soc Sci Med 52:1865-78, 2001
5. Roter DL: Patient participation in the patient-provider interaction: the effects of patient question asking on the quality of interaction, satisfaction and compliance. Health Educ Monogr 5:281- 315, 1977
6. Hebert RS, Schulz R, Copeland V, et al: What questions do family caregivers want to discuss with health care providers in order to prepare for the death of a loved one? An ethnographic study of caregivers of patients at end of life. J Palliat Med 11:476-83, 2008
7. Fitch MI: How much should I say to whom? J Palliat Care 10:90-100, 1994
8. Beisecker AE, Helmig L, Graham D, et al: Attitudes of oncologists, oncology nurses, and patients from a women's clinic regarding medical decision making for older and younger breast cancer patients. Gerontologist 34:505-12, 1994
9. Butow PN, Maclean M, Dunn SM, et al: The dynamics of change: cancer patients' preferences for information, involvement and support. Ann Oncol 8:857-63, 1997
10. Brown RF, Butow PN, Dunn SM, et al: Promoting patient participation and shortening cancer consultations: a randomised trial. Br J Cancer 85:1273-9, 2001
11. Bruera E, Pituskin E, Calder K, et al: The addition of an audiocassette recording of a consultation to written recommendations for patients with advanced cancer: A randomized, controlled trial. Cancer 86:2420-5,1999
12. Dunn SM, Butow PN, Tattersall MH, et al: General information tapes inhibit recall of the cancer consultation. J Clin Oncol 11:2279-85, 1993
13. Hogbin B, Fallowfield L: Getting it taped: the 'bad news' consultation with cancer patients. Br J Hosp Med 41:330-3, 1989
14. Tattersall MH, Butow PN, Griffin AM, et al: The take-home message: patients prefer consultation audiotapes to summary letters. J Clin Oncol 12:1305-11, 1994
15. O'Cathain A, Thomas KJ: Evaluating decision aids--where next? Health Expect 7:98-103, 2004
16. Bruera E, Sweeney C, Willey J, et al: Breast cancer patient perception of the helpfulness of a prompt sheet versus a general information sheet during outpatient consultation: a randomized, controlled trial. J Pain Symptom Manage 25:412-9, 2003
17. Glynne-Jones R, Ostler P, Lumley-Graybow S, et al: Can I look at my list? An evaluation of a 'prompt sheet' within an oncology outpatient clinic. Clin Oncol (R Coll Radiol) 18:395-400, 2006
18. Weiner JS, Roth J: Avoiding iatrogenic harm to patient and family while discussing goals of care near the end of life. J Palliat Med 9:451-63, 2006
19. Wetzels R, Wensing M, van Weel C, et al: A consultation leaflet to improve an older patient's involvement in general practice care: a randomized trial. Health Expect 8:286-94, 2005
20. Cegala DJ, Post DM, McClure L: The effects of patient communication skills training on the discourse of older patients during a primary care interview. J Am Geriatr Soc 49:1505-11, 2001

21. Fleissig A, Glasser B, Lloyd M: Encouraging out-patients to make the most of their first hospital appointment: to what extent can a written prompt help patients get the information they want? Patient Educ Couns 38:69-79, 1999

22. Paci E, Barneschi MG, Miccinesi G, et al: Informed consent and patient participation in the medical encounter: a list of questions for an informed choice about the type of anaesthesia. Eur J Anaesthesiol 16:160-5, 1999

23. Kidd J, Marteau TM, Robinson S, et al: Promoting patient participation in consultations: a randomised controlled trial to evaluate the effectiveness of three patient-focused interventions. Patient Educ Couns 52:107-12, 2004

24. Parker PA, Davison BJ, Tishelman C, et al: What do we know about facilitating patient communication in the cancer care setting? Psychooncology 14:848-58; discussion 859-60, 2005

25. Brown R, Butow PN, Boyer MJ, et al: Promoting patient participation in the cancer consultation: evaluation of a prompt sheet and coaching in question-asking. Br J Cancer 80:242-8, 1999

26. Clayton JM, Butow PN, Tattersall MHN, et al: Randomized controlled trial of a prompt list to help advanced cancer patients and their caregivers to ask questions about prognosis and end-of- life care. Journal of Clinical Oncology 25:715-723, 2007

27. Butow PN, Dunn SM, Tattersall MH, et al: Patient participation in the cancer consultation: evaluation of a question prompt sheet. Ann Oncol 5:199-204, 1994

28. Hebert RS, Schulz R, Copeland VC, et al: Pilot testing of a question prompt sheet to encourage family caregivers of cancer patients and physicians to discuss end-of-life issues. Am J Hosp Palliat Care 26:24-32, 2009

29. Yeh JC, Cheng MJ, Chung CH, et al: Using a question prompt list as a communication aid in advanced cancer care. J Oncol Pract 10:e137-41, 2014

30. Arthur J, Yennurajalingam S, Williams J, et al: Development of a Question Prompt Sheet for Cancer Patients Receiving Outpatient Palliative Care. J Palliat Med, 2016

31. Clayton JM, Natalia C, Butow PN, et al: Physician endorsement alone may not enhance question-asking by advanced cancer patients during consultations about palliative care. Support Care Cancer 20:1457-64,2012

32. Clayton JM, Butow PN, Tattersall MH, et al: Randomized controlled trial of a prompt list to help advanced cancer patients and their caregivers to ask questions about prognosis and end-of-life care. J Clin Oncol 25:715-23, 2007

33. Blanchard CG, Ruckdeschel JC, Fletcher BA, et al: The impact of oncologists' behaviors on patient satisfaction with morning rounds. Cancer 58:387-93, 1986

34. Ong LM, Visser MR, Lammes FB, et al: Doctor-patient communication and cancer patients' quality of life and satisfaction. Patient Educ Couns 41:145-56, 2000

35. Zandbelt LC, Smets EM, Oort FJ, et al: Satisfaction with the outpatient encounter: a comparison of patients' and physicians' views. J Gen Intern Med 19:1088-95, 2004

36. Spielberger CD: Manual for the State-Trait Anxiety Inventory (Form Y). Palo Alto, CA, Consulting Psychologist Press, 1983

37. Cassileth BR, Zupkis RV, Sutton-Smith K, et al: Information and participation preferences among cancer patients. Ann Intern Med 92:832-6, 1980

38. Degner LF, Sloan JA, Venkatesh P: The Control Preferences Scale. Can J Nurs Res 29:21- 43, 1997

39. Sutherland HJ, Llewellyn-Thomas HA, Lockwood GA, et al: Cancer patients: their desire for information and participation in treatment decisions. J R Soc Med 82:260-3, 1989

40. Kryworuchko J, Stacey D, Bennett C, et al: Appraisal of primary outcome measures used in trials of patient decision support. Patient Educ Couns 73:497-503, 2008

41. Davison BJ, Kirk P, Degner LF, et al: Information and patient participation in screening for prostate cancer. Patient Educ Couns 37:255-63, 199
