# Supplementary material for: Helpfulness of Question Prompt Sheet for Patient-Physician Communication Among Patients With Advanced Cancer: A Randomized Clinical Trial
Source: JAMA Netw Open. 2023 May 2;6(5):e2311189. doi: 10.1001/jamanetworkopen.2023.11189 (PMC10155065; doi:10.1001/jamanetworkopen.2023.11189)
Supplement: Supplement 2. — eAppendix 1. Question Prompt Sheet eAppendix 2. General Information Sheet eTable. Effect of Information Material on Participant Speaking Time, Consultation Duration, Questions Asked, Anxiety and Overall Satisfaction [file jamanetwopen-e2311189-s002.pdf]

Supplemental Online Content

Arthur J, Pawate V, Lu Z, et al. Helpfulness of question prompt sheet for patient-physician communication among patients with advanced cancer: a randomized clinical trial. *JAMA Netw Open*. 2023;6(5):e2311189. doi:10.1001/jamanetworkopen.2023.11189

eAppendix 1. Question Prompt Sheet

eAppendix 2. General Information Sheet

eTable. Effect of Information Material on Participant Speaking Time, Consultation Duration, Questions Asked, Anxiety and Overall Satisfaction

This supplemental material has been provided by the authors to give readers additional information about their work.

© 2023 Arthur J et al. *JAMA Network Open.*

eAppendix 1. Question Prompt Sheet

THE PALLIATIVE CARE SERVICE

1. Who are the members of the palliative care team and what do they do?
2. How do I access the services offered by the palliative care team?
3. How and when can I contact the palliative care team?
4. What is the role of my primary physician now that I have been referred to the palliative care team?

SYMPTOMS, TREATMENT AND LIFESTYLE

5. If I have symptoms, what can be done to improve them? (E.g. pain or discomfort, constipation, shortness of breath, nausea or feeling sick, lack of appetite, tiredness, dry mouth)?
6. What are the common side effects of my medications?
7. Are there any medications that I should STOP taking because of their interactions with the newly prescribed medication?
8. Can I stop taking the pain medication if my pain goes away?
9. What can I do if I am not coping?
10. Who can I talk to about the medical care that I want in the future when I am no long able to speak for myself?
11. What symptoms may occur in the future and what should I do if they arise?
12. How can I cope with the changes in my body as a result of this illness?

TYPES OF SUPPORT

13. Can someone help me to communicate with other members of my family about what is happening to me?
14. Is there someone I can talk to about my fears, concerns, spiritual or religious needs?
15. What support is available for other people in the family, such as my caregiver or my children?

END OF LIFE ISSUES ***(The following questions may not be relevant to you or your stage of illness. You may or may not want to read this section, but there may come a time in the future when you want to ask some of these questions.)***

16. How do I get my affairs in order and write a will?
17. How do I get information about advance directives?
18. How do I get information about hospice?
19. Is it feasible for me to die at home rather than in the hospital?
20. What can I expect in my last days of my life?

FOR CAREGIVERS

21. How do I get help if I am no l conger able to take care of my loved one?
22. What skills will I need as a caregiver?
23. How can I best support the person that I am caring for?
24. What should I say when the person that I am caring for asks, "am I dying"?
25. Will you be able to tell me when it is getting close to the time that he/she will die?

© 2023 Arthur J et al. *JAMA Network Open.*

eAppendix 2. General Information Sheet

THE PALLIATIVE CARE TEAM AND SERVICES

The palliative and supportive care team consists of specialized, highly trained members such as a physician, nurse, pharmacist, psychologist, counsellor, social worker, and if needed, a chaplain. They use conventional and complementary therapies to improve the quality of life for both patients and families and will attend to you according to your individual needs.

SYMPTOMS, TREATMENT AND LIFESTYLE

Palliative and supportive care is medical treatment aimed at improving the quality of life and relieving suffering for patients and their families faced with life threatening illnesses. We help with treating physical symptoms like pain, shortness of breath, nausea, and fatigue. We also help with emotional and spiritual needs such as stress, anxiety, depression, grief, and coping. We assist patients in making critical decisions about their care and provide support for family or caregivers. All patients with cancer at any stage of the disease can receive supportive care.

LOCATIONS AND CONTACT NUMBERS

To accommodate patient needs, we offer the services at two clinic locations for our patients. The main clinic is located in the main hospital building on the 11th floor, and a satellite clinic is in the Mays building on the 8th floor. If you have any concerns or questions, please feel free to call us on weekdays from 8am to 5 pm at 713-792-6072. After 5pm on weekdays and at any time on weekends, contact us at the on-call pager number 713-404-1275 and follow the voice instructions.

© 2023 Arthur J et al. *JAMA Network Open.*

eTable 1. Effect of Information Material on Participant Speaking Time, Consultation Duration, Questions Asked, Anxiety and Overall Satisfaction

	Mean (SD)			
**Characteristic**	**Total** 130 (100%)	**QPS** 63 (48.5%)	**GIS** 67 (51.5%)	***p-value***
**Patient/caregiver speaking time, mins**	9.00 (5.38)	7.95 (5.31)	9.95 (5.32)	0.06
**Physician speaking time, mins**	14.15 (7.44)	14.21 (8.28)	14.11 (6.66)	0.95
**Consultation duration, mins**	27.61 (13.72)	26.76 (13.26)	28.44 (14.22)	0.52
**Patient post-consultation STAI anxiety ***	0.02 (0.43)	-0.01 (0.32)	0.04 (0.51)	0.48
**Patient overall satisfaction**	94.45 (12.47)	95.01 (10.51)	93.90 (14.18)	0.63
**Total number of patient/caregiver questions asked**	5.20 (5.41)	5.22 (6.15)	5.18 (4.66)	0.96
**Type of question asked**				
Treatment	3.03 (4.10)	2.95 (4.24)	3.10 (3.99)	0.83
Physical symptoms	0.54 (0.89)	0.49 (0.86)	0.58 (0.92)	0.57
PC team and services provided	0.40 (0.94)	0.49 (1.13)	0.31 (0.72)	0.28
Lifestyle and Quality of life	0.32 (1.27)	0.40 (1.73)	0.25 (0.56)	0.52
End of life issues	0.06 (0.39)	0.10 (0.53)	0.03 (0.17)	0.34
Prognosis	0.04 (0.23)	0.03 (0.25)	0.04 (0.21)	0.75
Other	0.05 (0.30)	0.02 (0.13)	0.07 (0.40)	0.27

Abbreviations: QPS, question prompt sheet; GIS, general information sheet; SD, standard deviation; PC, palliative care; STAI, State Anxiety Inventory
* Adjusted for pre-consultation scores

© 2023 Arthur J et al. *JAMA Network Open.*
