# Supplementary material for: Helpfulness of Question Prompt Sheet for Patient-Physician Communication Among Patients With Advanced Cancer: A Randomized Clinical Trial
Source: JAMA Netw Open. 2023 May 2;6(5):e2311189. doi: 10.1001/jamanetworkopen.2023.11189 (PMC10155065; doi:10.1001/jamanetworkopen.2023.11189)
Supplement: Supplement 3. — Data Sharing Statement [file jamanetwopen-e2311189-s003.pdf]

Data Sharing Statement

Arthur. Helpfulness of Question Prompt Sheet for Patient-Physician Communication Among Patients With Advanced Cancer. *JAMA Netw Open*. Published May 02, 2023. doi:10.1001/jamanetworkopen.2023.11189

Data
Data available: No
